# Penetrating Wounds of the Abdomen*Some remarks made in a discussion of this subject before the Clinical Society of Maryland, December 20, 1895.

**Published:** 1896-02

**Authors:** Randolph Winslow

**Affiliations:** Baltimore, Md.


### PENETRATING WOUNDS OF THE ABDOMEN.*

By RANDOLPH WINSLOW, M.D.,
BALTIMORE, MD.

My attention was first called practically to penetrating wounds of the abdomen in July, 1881. On the 14th of July of that year a colored man was admitted to the University Hospital, with a pistol wound, the ball entering the left side of the abdomen at a point one and a half inches from the linea alba and three inches from the umbilicus, making a circular hole, with clean-cut edges, about the size of the end of the little finger, through which about an inch of omentum protruded. There was no hemorrhage, and but little pain, the temperature was normal, and the pulse 80, and full and strong. As soon as I could replace the omentum it was ligatured and the redundant portion cut off. The wound was slightly enlarged, and a drainage-tube introduced. Two ice bladders were applied to the abdomen and a grain of opium administered every three hours. On the third day his temperature rose from 92 in the morning to $101\frac{3}{5}$ in the evening; the pulse from 80 to 126; and the respiration to 32; his abdomen became tympanitic, but there was very little tenderness on pressure. The onset of peritonitis was feared, but the next morning the temperature dropped to $99\frac{4}{5}$, and

*Some remarks made in a discussion of this subject before the Clinical Society of Maryland, December 20, 1895.

the pulse to 104, and subsequently no alarming symptoms occurred, and the man was discharged from the hospital in thirty days entirely well. This case came under my care just previous to the publication of Dr. J. Marion Sims's famous article advocating laparotomy in this class of injuries, and the favorable result is to be attributed to good fortune rather than to good treatment. It is evident that the ball spent itself in penetrating the abdominal wall and did not wound the intestines.

CASE 2. Pistol wound of epigastrium. June 3, 1894, S. P., negress, was shot with a large revolver, the bullet entering the epigastrium one and a half inches from the linea alba, and three inches below the ensiform cartilage. From the situation of the wound it was thought that the stomach or large intestine was injured, but there was no vomiting of blood nor bloody stools. There was some pain but no elevation of the temperature, and she was let alone. She lived five days and two hours, and died with symptoms suggestive of peritonitis. At the autopsy no peritonitis was found, nor any wound of the abdominal viscera. Some fluid was found in the peritoneal cavity, and the peritoneum detached, with an enormous blood-clot under it. A long slit was found in the aorta below the celiac axis and a counter opening opposite the third lumbar vertebra. There were thus two large openings in the aorta, and yet she lived five days and two hours.

CASE 3. Pistol wound of small intestine. Twenty-seven holes in intestines and bladder; death in eighteen hours. C. W., admitted on same day as above, was shot in the hypogastric region, over the pubes, with a large pistol. One wound was in the hypogastrium and a bullet was cut out from the integument one inch distant. Another wound was found upon the buttock, near the anus, and a bullet was removed from the skin three inches distant. Was admitted to the hospital during the night. Pulse good and shock not marked; urine bloody. When seen by me the next day was in collapse, pulseless, vomiting, and in great pain. Autopsy: The bullets were found to have pursued nearly parallel courses, one entering in front and lodging under the skin of the buttock; the other entering behind and lodging beneath the skin of the abdomen. The peritoneal cavity was filled with blood, feces, pepperpods and cherry-stones; the lower portion of the small intestine, for about six feet, was riddled, there being twenty-five holes in the bowel, one in the mesentery, one in the top of the bladder and one in the base ; altogether twenty-six wounds as the result of these two bullets.

Laparotomy should have been performed in both of these cases, though there is scarcely a chance that a favorable result could have been obtained. I only saw the last case when he was in collapse.

CASE 4. Pistol wound of liver; death in eighteen or twenty hours. D. G., colored ; was shot on the same day as the two preceding cases. The wound was on the right side, in the anterior axillary line, about two inches from the nipple, in the fifth intercostal space, and the ball ranging downwards fractured the sixth and seventh ribs. Much bleeding and marked shock followed. Local pain and pain in the right shoulder. There was no escape of bile from the wound. The patient vomited the contents of the stomach, but no blood. A wound of the liver was diagnosticated. The patient never rallied from the shock, and died in the evening of the same day. Autopsy : The bullet was found to have passed through the upper surface of the liver and to be imbedded in the substance of the diaphragm.

CASE 5. Pistol wound of back, perforation of small intestine, death in twenty-nine hours, from peritonitis. W. G., colored, was shot whilst running away from a policeman, the bullet entering his back one inch in the left of the second lumbar vertebra, and a probe could not be made to follow the track. Seven hours after injury the pulse was 82, respiration 32, temperature 96.8, and there was no shock. Soon pain about the umbilicus set in, and rigidity of the abdominal muscles, vomiting, and a bloody alvine dejection, and there was numbness of the parts supplied by the left anterior crural nerve. The temperature began to rise and in twelve hours reached 100.2, pulse 90, respiration 48 and thoracic in character, the abdominal tenderness remaining. A consultation was held in regard to the propriety of performing laparotomy in this case, but the consultant was opposed to it. The patient died of peritonitis in twenty-nine hours. Autopsy : The ball entered the back opposite the second lumbar vertebra, grazed the third lumbar vertebra, then passed into the peritoneal cavity, pierced the small intestine in two places, and finally dropped into the pelvis. Feces had escaped and an intense general peritonitis was set up. Laparotomy ought to have been performed in this case.

CASE 6. Gunshot wound, supposed to have penetrated the abdominal cavity. Operation declined. Recovery. Michael Kane, age forty-five, admitted December 2, 1893. He was shot a short time before admission, with a pistol, the bullet entering three inches to the left of the sternum and one inch below, just over the left costal margin. Dr. Spruill examined him under an anesthetic and thought the ball had gone into the peritoneal cavity. After examining the man I recommended an exploratory laparotomy, which he declined. No serious symptoms supervened, and he left the hospital on December 20th. The highest temperature was 101 and pulse 80.

After a consideration of these cases I believe that a person who comes into contact with a case of penetrating wound of the abdomen has not done his duty if he fails to give the patient an opportunity for life by performing section. A certain number of cases will recover without operation, but such treatment is simply working in the dark, and in those cases it is probable that the viscera have not been injured. If they have been injured it is almost inevitable that death will follow. No surgical rule is better established than that such wounds should be treated by an exploring section, and, if necessary, by the sewing up of the wounds and ligation of vessels that have been ruptured. There are many points in regard to the technique of such operations, which I will not now take up your time in considering, but I wish again to express my opinion in thorough conformity with what has been said, that a surgeon is not justified in permitting a patient to go without laparotomy who has received a penetrating wound of the abdomen. There is but one thing to do in these cases, and that is to perform laparotomy and be further guided by the circumstances of the case.

